# Associational Resistance to Predation by Protists in a Mixed Species Biofilm

**DOI:** 10.1128/aem.01741-22

**Published:** 2023-01-19

**Authors:** Yu Fen Goh, Henriette L. Røder, Siew Herng Chan, Muhammad Hafiz Ismail, Jonas S. Madsen, Kai Wei Kelvin Lee, Søren J. Sørensen, Michael Givskov, Mette Burmølle, Scott A. Rice, Diane McDougald

**Affiliations:** a Singapore Centre for Environmental Life Sciences Engineering, Nanyang Technological University, Singapore, Singapore; b Section of Microbiology, Department of Biology, University of Copenhagen, Copenhagen, Denmark; c Interdisciplinary Graduate School, Nanyang Technological University, Singapore, Singapore; d Department of Immunology and Microbiology, University of Copenhagen, Copenhagen, Denmark; e Microbiomes for One Systems Health and Agriculture and Food, CSIRO, Westmead NSW, Australia; f Australian Institute for Microbiology and Infection, University of Technology Sydney, Sydney, Australia; Shanghai Jiao Tong University

**Keywords:** *Pseudomonas aeruginosa*, *Klebsiella pneumoniae*, *Pseudomonas protegens*, protozoa, predation, multispecies biofilm, protection

## Abstract

Mixed species biofilms exhibit increased tolerance to numerous stresses compared to single species biofilms. The aim of this study was to examine the effect of grazing by the heterotrophic protist, Tetrahymena pyriformis, on a mixed species biofilm consisting of Pseudomonas aeruginosa, Pseudomonas protegens, and Klebsiella pneumoniae. Protozoan grazing significantly reduced the single species K. pneumoniae biofilm, and the single species P. protegens biofilm was also sensitive to grazing. In contrast, P. aeruginosa biofilms were resistant to predation. This resistance protected the otherwise sensitive members of the mixed species biofilm consortium. Rhamnolipids produced by P. aeruginosa were shown to be the primary toxic factor for *T. pyriformis.* However, a rhamnolipid-deficient mutant of P. aeruginosa (P. aeruginosa Δ*rhlAB*) maintained grazing resistance in the biofilm, suggesting the presence of at least one additional protective mechanism. P. aeruginosa with a deleted gene encoding the type III secretion system also resisted grazing. A transposon library was generated in the Δ*rhlAB* mutant to identify the additional factor involved in community biofilm protection. Results indicated that the Pseudomonas Quinolone Signal (PQS), a quorum sensing signaling molecule, was likely responsible for this effect. We confirmed this observation by showing that double mutants of Δ*rhlAB* and genes in the PQS biosynthetic operon lost grazing protection. We also showed that PQS was directly toxic to *T. pyriformis*. This study demonstrates that residing in a mixed species biofilm can be an advantageous strategy for grazing sensitive bacterial species, as P. aeruginosa confers community protection from protozoan grazing through multiple mechanisms.

**IMPORTANCE** Biofilms have been shown to protect bacterial cells from predation by protists. Biofilm studies have traditionally used single species systems, which have provided information on the mechanisms and regulation of biofilm formation and dispersal, and the effects of predation on these biofilms. However, biofilms in nature are comprised of multiple species. To better understand how multispecies biofilms are impacted by predation, a model mixed-species biofilm was here exposed to protozoan predation. We show that the grazing sensitive strains *K. pneumonia* and *P. protogens* gained associational resistance from the grazing resistant P. aeruginosa. Resistance was due to the secretion of rhamnolipids and quorum sensing molecule PQS. This work highlights the importance of using mixed species systems.

## INTRODUCTION

Grazing by protozoa is one of the main mortality factors for bacteria in natural environments (1), and thus predation plays an important role in shaping bacterial communities (2). Protozoan grazing exerts a strong selective pressure on bacteria to develop anti-predator adaptations such as the formation of bacterial aggregates and grazing resistant microcolonies (3, 4). For example, the suspension feeder *Cafeteria roenbergensis* effectively feeds on planktonic cells of Vibrio cholerae, while biofilms are resistant to predation by surface feeding protozoa (5). In addition, protozoan grazing has been shown to activate the secretion of antiprotozoal compounds, which were also effective against amoeba and flagellates (4–7).

Biofilms are complex communities organized in a self-produced extracellular polymeric substance matrix that protect biofilm cells from a variety of stresses, such as antimicrobial agents (8) and grazing by protists (1, 6, 9). Biofilms in the environment exist as mixed species communities that are known to display a higher level of tolerance to various stresses than single species biofilms (10–12). P. K. Raghupathi et al. (13) discovered that a multispecies biofilm of four bacterial isolates from soil, *Xanthomonas retroflexus*, Stenotrophomonas rhizophila, Microbacterium oxydans, and Paenibacillus amylolyticus, exposed to predation by the ciliate Tetrahymena pyriformis, produced more biofilm than nongrazed biofilms. Hence, the ubiquity of mixed species biofilms and their ability to resist various stresses highlights the importance of investigating the influence of protozoan grazing on these biofilms.

Predation resistance of Pseudomonas aeruginosa biofilms has been demonstrated to be due to both chemical and physical defenses. When exposed to the amoeba Acanthamoeba castellanii, P. aeruginosa was shown to induce the type III secretion system, which resulted in lysis of the predator (14). In addition, in the presence of the flagellate *Rhynchomonas nasuta*, undifferentiated early biofilms of P. aeruginosa formed microcolonies that were protected from predation (4).

In the present study, we examined the grazing resistance of both single and mixed Pseudomonas protegensspecies biofilms consisting of P. aeruginosa (PAO1), K. pneumoniae (KP-1), and P. protegens (Pf-5) to the heterotrophic ciliate *T. pyriformis*, a ciliate that feeds both on planktonic cells and early biofilms. Our results demonstrated that the grazing sensitive bacteria K. pneumoniae and P. protegens benefited from being in a mixed biofilm with P. aeruginosa. The ability of P. aeruginosa to protect both mono and mixed species biofilms was examined to further elucidate the underlying resistance mechanism(s). Our results show that P. aeruginosa produces a secreted compound, rhamnolipids, as well as a biofilm associated compound, PQS, that is responsible for the shared protection of the mixed species community.

## RESULTS

### Effect of predation on single and mixed species biofilms.

**FIG 1:**
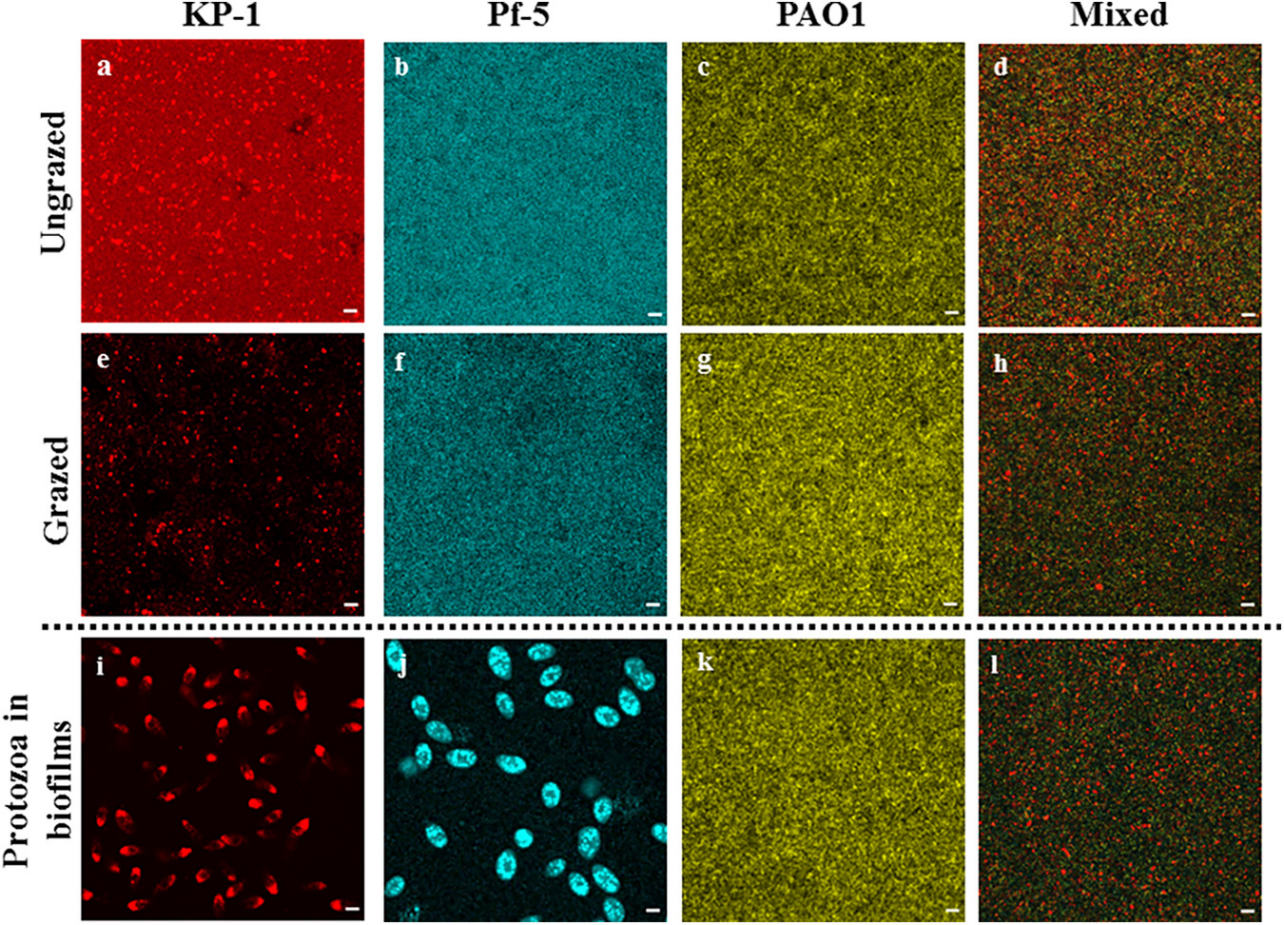
Confocal micrographs of ungrazed and grazed single and mixed species biofilms formed by P. aeruginosa (PAO1 WT, yellow), P. protegens (Pf-5, cyan) and K. pneumoniae (KP-1, red). Biofilms were pregrown for 48 h, at which point either nothing or *T. pyriformis* was added and the biofilms were incubated for a further 48 h (96 h total). Panels (a to h) show the difference in biomass between the ungrazed and grazed single and mixed species biofilms and (i to l) panels show the presence or absence of *T. pyriformis* after grazing. No *T. pyriformis* were observed in the mixed or single species P. aeruginosa biofilms. Scale bar: 20 μm.

Predator-prey interactions between *T. pyriformis* and single and mixed species biofilms of wild type P. aeruginosa, P. protegens, and K. pneumoniae were examined by coincubation for 48 h. Confocal imaging of the grazed biofilms of K. pneumoniae revealed that there was approximately a 9-fold reduction in biomass compared to the ungrazed biofilm (Fig. 1a and e). Red fluorescence observed inside the *T. pyriformis* indicated that the loss of biofilm biomass was due to *T. pyriformis* grazing on the bacterium (Fig. 1I). Although there was no observable reduction in biomass of the grazed biofilms of P. protegens (Fig. 1b and f), cyan fluorescence within *T. pyriformis* food vacuoles indicated that the biofilm was being grazed (Fig. 1j). In contrast to the K. pneumoniae and P. protegens biofilms, single species biofilms of P. aeruginosa (Fig. 1c and g) and the mixed species biofilms (Fig. 1d and h) were unaffected by grazing, and thus predation resistant. Furthermore, no protozoa were observed on either biofilm containing P. aeruginosa (Fig. 1k and l), indicating that these biofilms were toxic to the predator.

**FIG 2:**
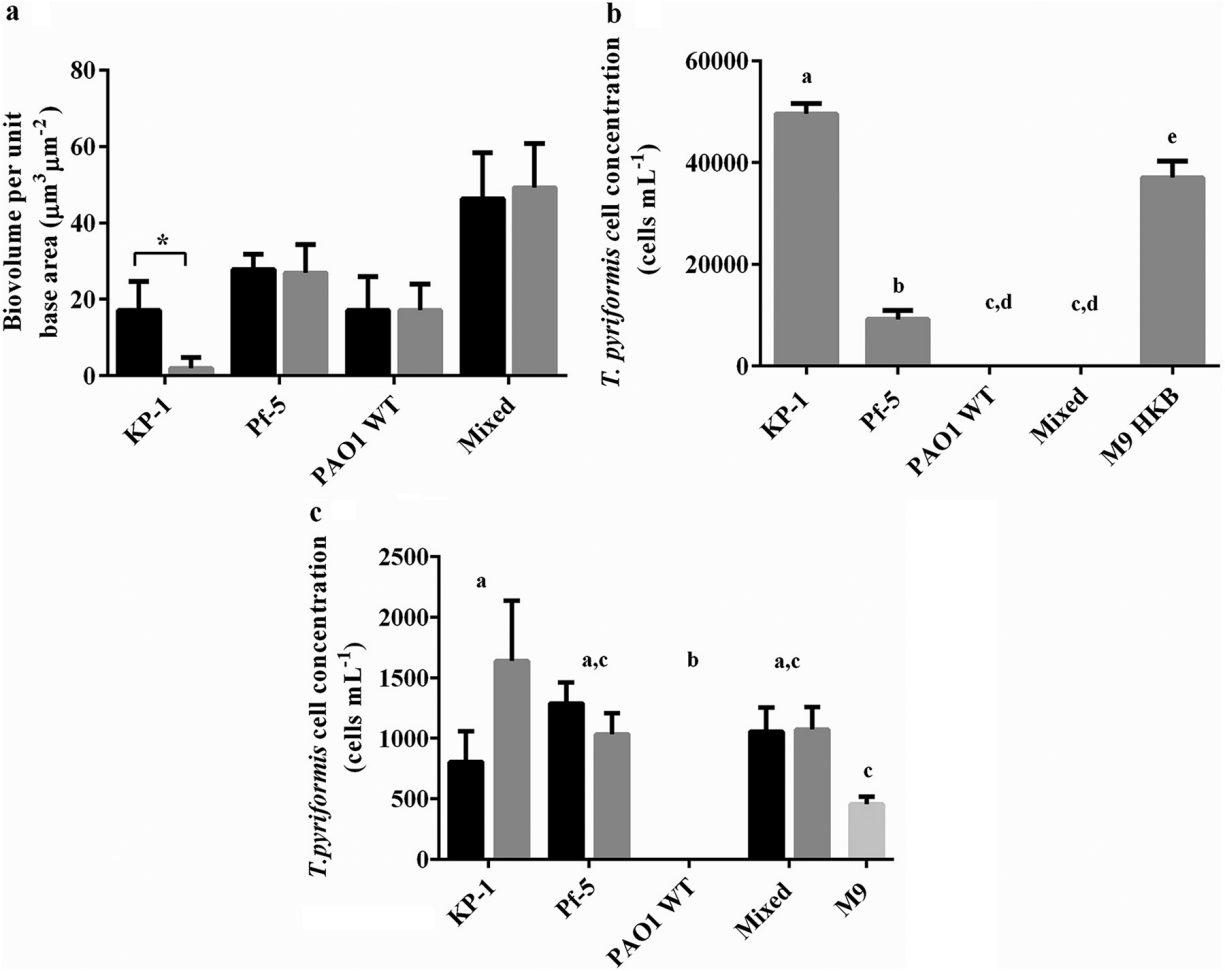
Predation of single and mixed species biofilms by *T. pyriformis*. (a) Biovolume per unit base area (μm^3^ μm^−2^) of single and mixed species ungrazed (black bars) and grazed (gray bars) biofilms after 48 h. (b) Enumeration of *T. pyriformis* in the grazed biofilms and M9 media controls with 10^8^ cells mL^−1^ of heat killed bacteria (HKB). (c) Enumeration of *T. pyriformis* exposed to cell-free supernatants from single and mixed species biofilms. Ungrazed (black bars) and grazed (gray bars) biofilms after 48 h. (*n* = 3). *, *P* < 0.05. The different letters are used to indicate significant differences between groups (*P* < 0.05).

One-way analysis of variance (ANOVA) of the biofilm images confirmed that there were no significant differences in the biovolumes of ungrazed and grazed biofilms of P. aeruginosa, P. protegens, or the mixed species biofilm (Fig. 2a). Quantitative image analysis of the mixed species biofilm indicated that it consisted of 13% K. pneumoniae, 47% P. protegens and 40% P. aeruginosa (Fig. S1). There was no significant difference in the proportion of P. aeruginosa, P. protegens, or K. pneumoniae in the ungrazed and grazed mixed species biofilms. Enumeration of *T. pyriformis* coincubated with the P. protegens and K. pneumoniae biofilms revealed there were 9170 ± 678 and 49,600 ± 227 cells mL^−1^, respectively (Fig. 2b). In contrast, no *T. pyriformis* remained after coincubation with the P. aeruginosa or mixed species biofilms, indicating toxicity of these biofilms toward the predator.

### Toxicity of cell-free supernatants to T. pyriformis.

To test for the presence of a secreted toxin that affects the survival of *T. pyriformis*, cell-free supernatants from single and mixed species biofilms were collected and added to cultures of *T. pyriformis*. Exposure to supernatants from ungrazed or grazed P. aeruginosa biofilms resulted in the death of the protist (Fig. 2c). In contrast, supernatants obtained from the mixed species biofilms containing P. aeruginosa did not demonstrate toxicity toward *T. pyriformis* (Fig. 2c). Enumeration of ciliates exposed to single species P. protegens or K. pneumoniae supernatants indicated that those supernatants were not toxic to *T. pyriformis* (Fig. 2c).

### The effect of rhamnolipids on the predation resistance of biofilms.

**FIG 3:**
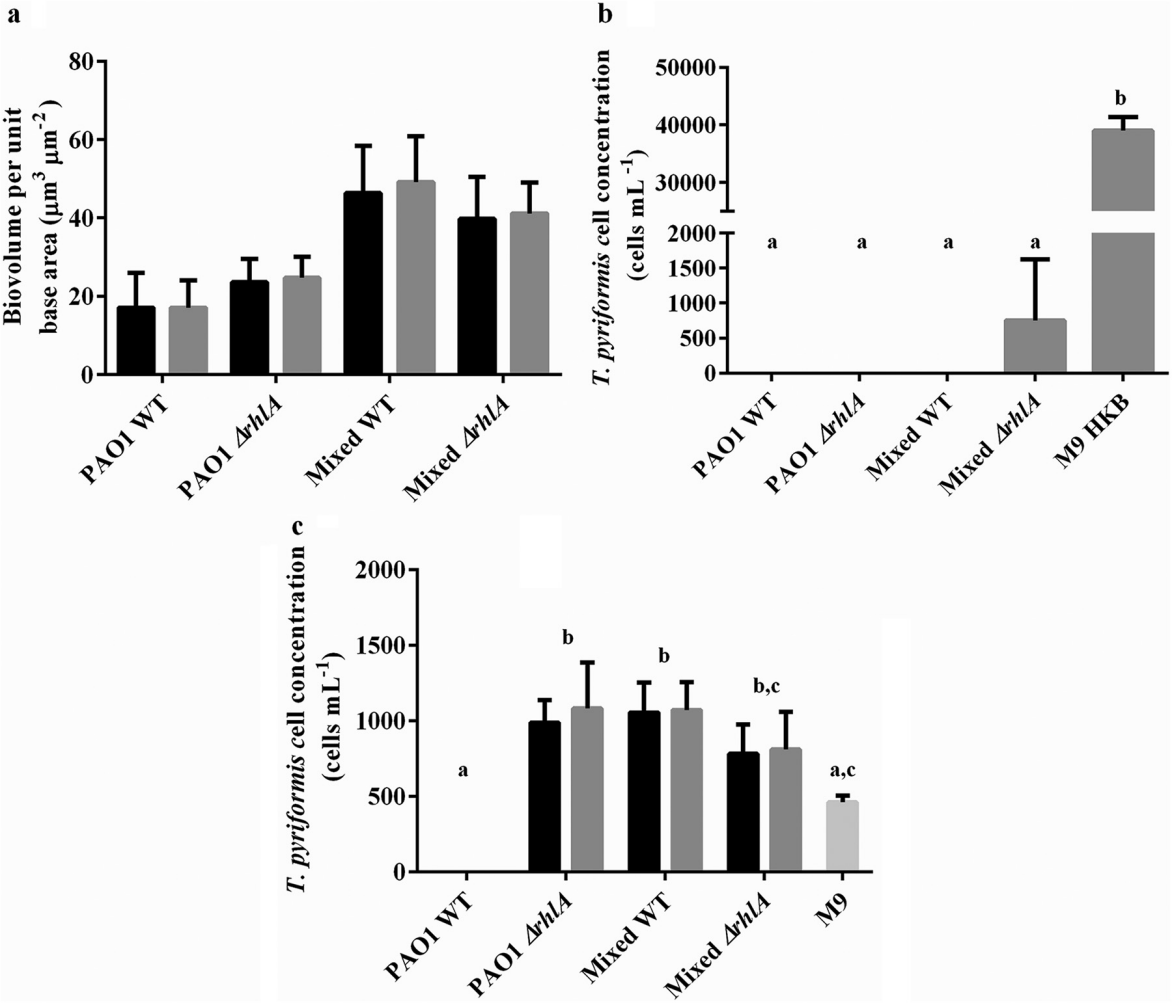
*T. pyriformis* predation on single and mixed species biofilms containing the P. aeruginosa *ΔrhlA* mutant. (a) Biovolume per unit base area (μm^3^ μm^−2^) of ungrazed (black bars) and grazed (gray bars) biofilms after 48 h. (b) The number of *T. pyriformis* after coincubation with biofilms and M9 media controls with 10^8^ cells mL^−1^ of heat killed bacteria (HKB). (c) Enumeration of *T. pyriformis* exposed to cell-free supernatants from single and mixed species biofilms. Ungrazed (black bars) and grazed (gray bars) biofilms after 48 h. The different letters are used to indicate significant differences between groups (*P* < 0.05).

The toxicity of the cell-free supernatants from P. aeruginosa biofilms indicated that the anti-protozoan activity was due to a secreted factor. It is known that rhamnolipids are secreted by P. aeruginosa biofilms; thus, to determine if rhamnolipid production was the anti-protozoal compound, *T. pyriformis* was exposed to a biofilm of a P. aeruginosa *ΔrhlA* strain that is defective for rhamnolipid production. Results show that despite the lack of rhamnolipid production, the biovolumes of ungrazed and grazed single species biofilms of P. aeruginosa *ΔrhlA* were not significantly different (Fig. 3a), indicating that these biofilms were resistant to predation. In contrast to the single species P. aeruginosa *ΔrhlA* biofilm, the mixed species biofilm containing P. aeruginosa *ΔrhlA* did not exhibit the same level of toxicity, as *T. pyriformis* was observed in the mixed biofilm (750 ± 876 cells mL^−1^ after coincubation for 48 h, Fig. 3b). The P. aeruginosa *ΔrhlA* mutant strain comprised only 22% of the biofilm biomass in the mixed biofilm, which was significantly different compared to 39% for the WT strain (Fig. S1).

Cell-free supernatants obtained from both P. aeruginosa *ΔrhlA* ungrazed and grazed biofilms were less toxic to *T. pyriformis* than those of the wild-type P. aeruginosa (Fig. 3c), indicating that rhamnolipids do confer grazing resistance to the single species biofilms. Interestingly, the single species biofilm of P. aeruginosa *ΔrhlA* was still grazing resistant despite the loss of rhamnolipid production (Fig. 3a), suggesting that there is another mechanism for predation resistance expressed by P. aeruginosa *ΔrhlA* biofilms.

### Role of type III secretion system in predation resistance.

**FIG 4:**
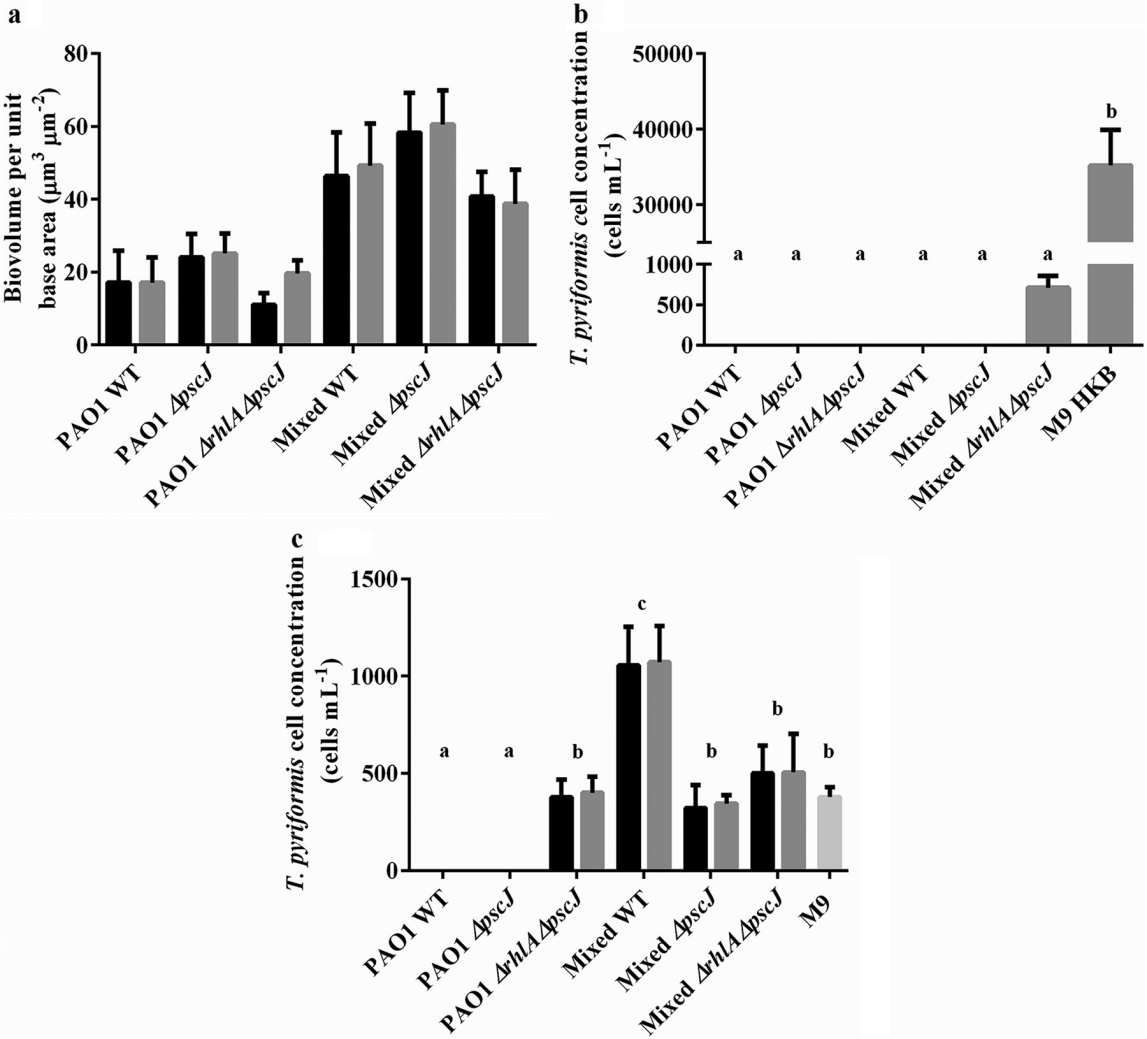
*T. pyriformis* predation of single and mixed species biofilms formed by P. aeruginosa *ΔpscJ* and *ΔrhlA ΔpscJ* strains. (a) Biovolume per unit base area (μm^3^ μm^−2^) of ungrazed (black bars) and grazed (gray bars) biofilms after 48 h. (b) Enumeration of *T. pyriformis* in biofilms and M9 media controls with 10^8^ cells mL^−1^ of heat killed bacteria (HKB). (c) Enumeration of *T. pyriformis* exposed to cell-free supernatants from single and mixed species biofilms. Ungrazed (black bars) and grazed (gray bars) biofilms after 48 h. (*n* =3). The different letters are used to indicate significant differences between groups (*P* < 0.05).

It has previously been reported that the type III secretion system was important for protecting late stage P. aeruginosa biofilms from predation by amoebae (14). Hence, a mutant defective in the type III secretion system (*pscJ*) and a double mutant in *rhlA* and *pscJ* were constructed and their grazing resistance in single and mixed species biofilms tested. One-way analysis of variance (ANOVA) comparisons of the biovolumes of the ungrazed and grazed biofilms indicated no significant differences (Fig. 4a). No *T. pyriformis* were detected after coincubation with the single or mixed species biofilms of the *pscJ* mutant (Fig. 4b). Similarly, there were no viable *T. pyriformis* in the P. aeruginosa *ΔrhlA ΔpscJ* single species biofilms. However, when P. aeruginosa *ΔrhlA ΔpscJ* was grown as part of a mixed species biofilm, *T. pyriformis* was observed in the biofilm (749 ± 150 cells mL^−1^). Furthermore, filtered supernatants obtained from the P. aeruginosa *ΔrhlA ΔpscJ* single and mixed species biofilms were not toxic toward *T. pyriformis*. In contrast, single species biofilms of P. aeruginosa *ΔpscJ* remained toxic toward *T. pyriformis* (Fig. 4c), probably be due to the production of rhamnolipids by the *pscJ* mutant.

The proportion of P. aeruginosa *ΔpscJ* in the mixed species biofilm consortia was 37%, which was similar to the proportion of P. aeruginosa wild type in the mixed species biofilms (39%). In contrast, the proportion of the P. aeruginosa *ΔrhlA ΔpscJ* strain was 13% in the mixed species biofilms compared to 22% and 39% for the P. aeruginosa *ΔrhlA* and P. aeruginosa WT strains, respectively (Fig. S1).

### Toxicity of purified rhamnolipids for *T. pyriformis*.

The data suggest that rhamnolipids may represent an important secreted factor that kills the predator. Therefore, the toxicity of rhamnolipids toward *T. pyriformis*, the minimum inhibition concentration (MIC) of rhamnolipids for *T. pyriformis,* was determined and found to be 0.03 g L^−1^ (Fig. S2). At this concentration of rhamnolipids, the *T. pyriformis* cells swelled and lysed immediately upon contact.

### Quantification of rhamnolipids produced by biofilms.

**FIG 5:**
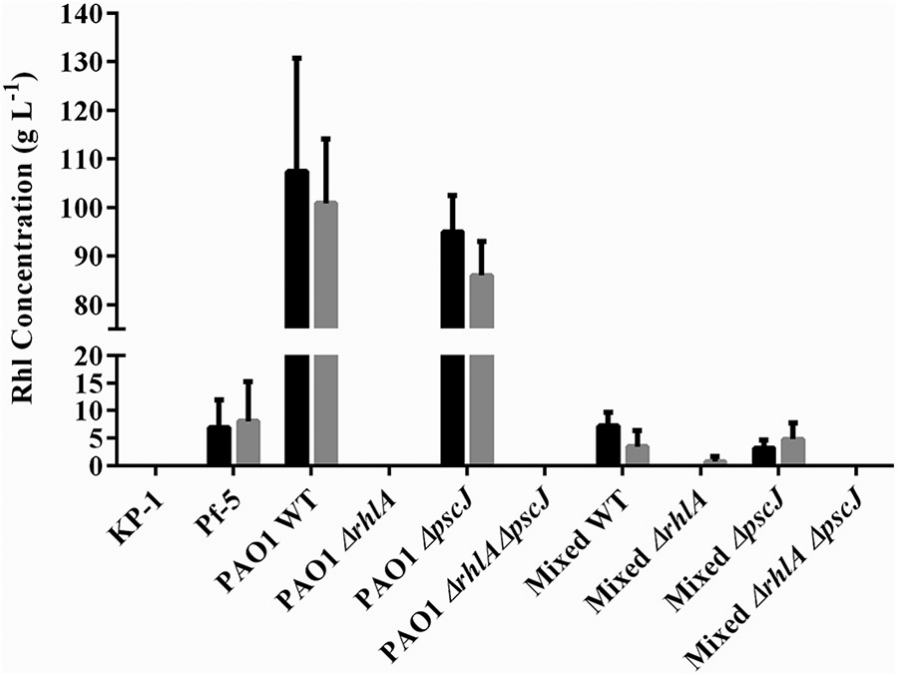
Rhamnolipid quantification from single and mixed species biofilms. Biofilms were pregrown for 48 h, at which time, either nothing was added (nongrazed controls, gray bars) or *T. pyriformis* was added and the biofilms were incubated for a further 48 h (96 h total). The concentration of rhamnolipids produced by the biofilms was subsequently quantified by orcinol assays (*n* = 3). No significant differences were found between the grazed and ungrazed biofilms.

The amount of rhamnolipids produced by the single and mixed species biofilms were quantified using the orcinol assay (15). No rhamnolipids were detected in the biofilms containing P. aeruginosa *ΔrhlA*, except for the mixed species biofilm, where trace quantities were detected (Fig. 5). Mixed species biofilms formed with either P. aeruginosa *ΔpscJ* or WT strains produced rhamnolipids ranging from approximately 5 to 8 g L^−1^ (Fig. 5), which was 100- to 200-fold higher than the MIC determined above. The levels of rhamnolipid did not differ significantly between grazed and ungrazed biofilms, suggesting that rhamnolipid production was not induced by the presence of the predator. The amount of rhamnolipids produced in the single species biofilms was at least 10-fold greater than in the mixed species biofilms. While single species biofilms of K. pneumoniae did not produce any rhamnolipids, P. protegens biofilms produced 6.94 ± 5.12 g L^−1^ and 8.03 ± 8.33 g L^−1^ of rhamnolipids in the ungrazed and grazed biofilms, respectively, and hence may account for the small amounts of rhamnolipids detected in the mixed species biofilms with the P. aeruginosa *rhlA* mutant.

### Screening of P. aeruginosa ΔrhlA transposon mutants for toxicity.

**FIG 6:**
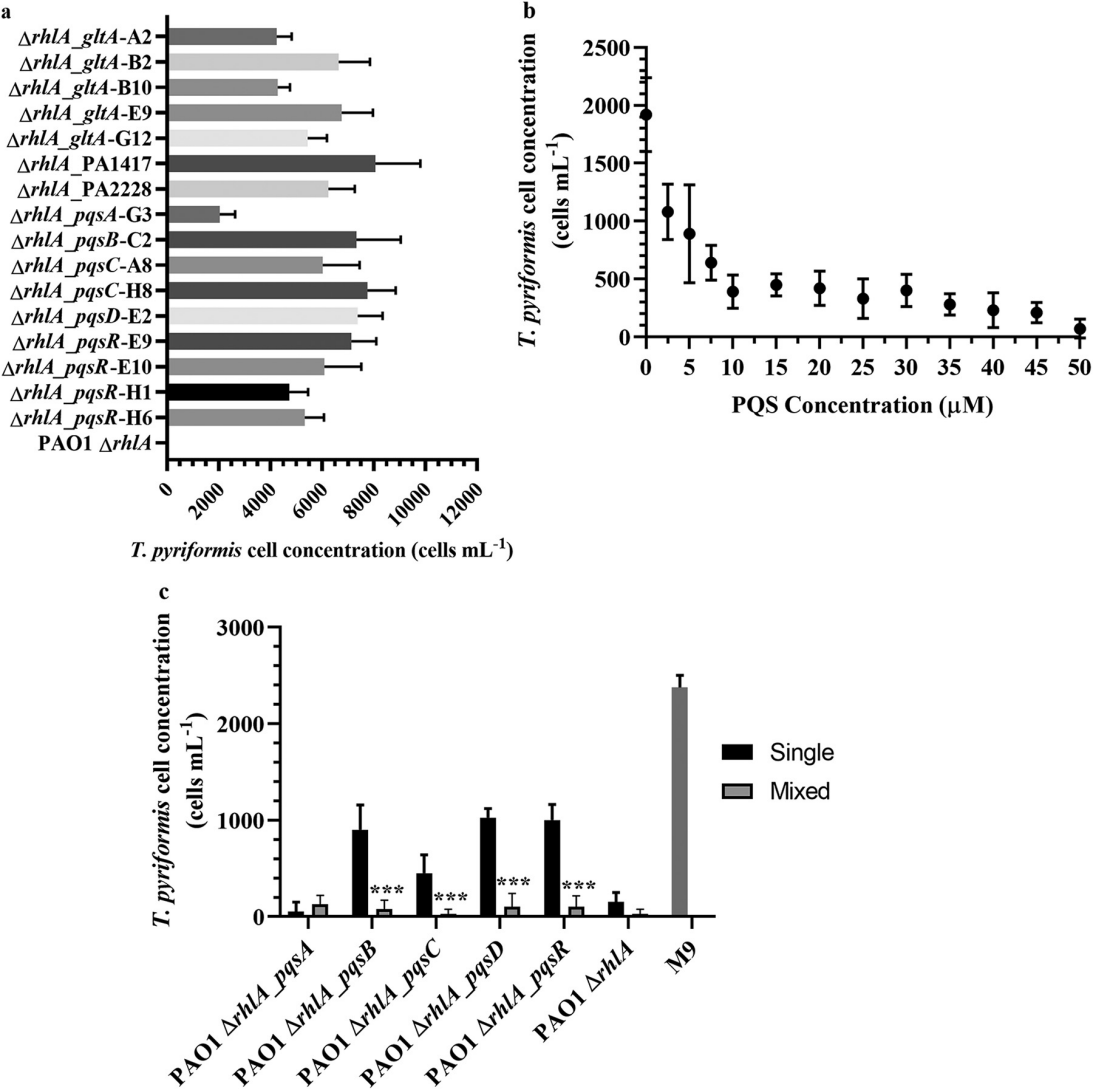
(a) Tetrahymena pyriformis survival in the biofilms of P. aeruginosa Δ*rhlA* transposon after 24 h of incubation. P. aeruginosa Δ*rhlA* transposon mutants are named according to the transposon insertion followed by the sample number. (b) Protozoan survival after exogenous addition of PQS. *T. Pyriformis* were exposed to different PQS concentrations dissolved in methanol after incubating for 2 h in M9 medium with heat killed bacteria. The maximum concentration of methanol was 1% of the total M9 volume. M9 medium containing 1% methanol without any PQS was used as the negative control. (c) *T. pyriformis* enumeration after 2 h in the biofilms of P. aeruginosa Δ*rhlA* transposon mutant biofilms with 50 μM PQS. Heat-killed bacteria with methanol but no PQS was used as a control. ***, *P* < 0.001. Differences were tested between mixed and single species biofilms. The number of protozoa was determined using light microscopy Data points represent the mean ± standard deviation of the mean (SD) (*n* = 3).

To identify the putative contact dependent killing factor, a P. aeruginosa Δ*rhlA* library of approximately 4,000 transposon mutants was tested for growth in M9 medium and mutants that could form biofilms in M9 medium were then screened for protozoan toxicity. In total, 16 mutants lacked toxicity to protozoa and were sequenced to identify the transposon insertion sites (Fig. 6). Five P. aeruginosa Δ*rhlA* mutants had transposon insertions in the citrate synthase gene, *gltA*. This was likely due to the carbon source, citrate, being used to select against the E. coli donor during the generation of the transposon mutants, thus allowing citrate synthase deficient mutants to survive. In addition, transposon insertions into the Pqs pathway were detected, including insertions in *pqsA*, *pqs B*, *pqs C*, *pqsD,* and *pqsR* (Table 1). This suggested that the Pqs pathway plays a role in regulating protozoa toxicity in the P. aeruginosa Δ*rhlA* mutant.

**TABLE 1:** Genomic locations of the inserted transposon

Gene	Coding position/total nt^*a*^	Product
*pqsA*	1518^*b*^1520/1554	PqsA
*pqsB*	40^*b*^41/852	PqsB
*pqsC*	472^*b*^473/1047	PqsC
	792^*b*^794/1047	
*pqsD*	84^*b*^85/1014	3-oxoacyl-[acyl-carrier-protein] synthase III
*pqsR*	166^*b*^167/999	Transcriptional regulator
	341^*b*^342/999	
	802^*b*^803/999	

a nt, total number of nucleotides in the gene.

b Indicates the nucleotide position where the transposon inserted in the gene.

### Exogeneous addition of PQS restores toxicity.

Given that the transposon data suggested that PQS may play a role in the secondary mechanism of killing *T. pyriformis*, the toxicity of PQS was evaluated by adding different concentrations of PQS to M9 medium containing P. aeruginosa Δ*rhlA* and incubating for 2 h before counting the number of viable protozoa. An initial addition of 1,000 protozoa was added to the medium, with the negative control containing 1% methanol without PQS (vol/vol). There was a 36% decrease in protozoal numbers in the presence of 7.5 μM PQS, where only 640 ± 151 protozoa survived compared to the initial 1,000 protozoa (Fig. 6b). As the concentration of PQS increased from 10 to 45 μM, the number of protozoa decreased from 390 ± 145 to 210 ± 88.0. At the highest concentration of 50 μM PQS, an average of 70.0 ± 82.0 protozoa remained in the biofilm after 2 h of incubation (Fig. 6b). This was comparable to the P. aeruginosa Δ*rhlA* biofilm with a PQS concentration of 42.9 μM as quantified by LC-MS where no protozoa were observed after 2 h, suggesting that PQS is toxic to protozoa at those concentrations.

The exogeneous addition of PQS (50 μM) to the single species biofilm of the P. aeruginosa Δ*rhlA* Δ*pqsA* transposon mutant restored toxicity, where only 50.0 ± 100 protozoa remained after 2 h of incubation, which was not significantly different from the positive control, P. aeruginosa Δ*rhlA* (Fig. 6c). The number of protozoa observed when incubated with the PqsB, PqsC, PqsD, and PqsR mutants in the presence of PQS was significantly lower than the control consisting of protozoa incubated with heat-killed bacteria in M9 medium. This further showed that the addition of PQS could restore toxicity against the protozoa (Fig. 6c). Surprisingly, PQS exhibited greater killing effect in all the mixed species biofilms than in the monospecies biofilms formed by the P. aeruginosa Δ*rhlA* transposon mutants, suggesting that the mixed species community might have contributed to the protozoa toxicity, although why this is the case requires further investigation to elucidate.

P. aeruginosa Δ*rhlA* quorum sensing mutants were measured for biofilm formation through crystal violet staining. Only *pqsC* mutant had comparable biofilm to that of P. aeruginosa Δ*rhlA.* The *pqsA*, *pqsD*, and *pqsR* mutants formed slightly lower number of biofilms while the *pqsB* mutant had a substantial reduction in biofilm formation (Fig. S4). Thus, for all strains except for the *pqsB* mutant, a difference in the amount of biofilm does not appear to account for the changes in protozoan killing.

## DISCUSSION

Biofilms typically exist as mixed species consortia, which can lead to increased biofilm biomass. When P. aeruginosa, P. protegens, and K. pneumoniae were grown together as a mixed species biofilm, the resulting biomass was greater than the sum of the single species biofilms, which agrees with our previous observations from this (11) and other (16, 17) consortia. Mixed species biofilms have also been found to have an increased resistance to a variety of stresses compared to single species biofilms (10, 11, 18–20). Here, we investigated the ability of a mixed species microbial community containing P. aeruginosa, P. protegens and K. pneumoniae to withstand protozoan grazing by the filter-feeding ciliate, *T. pyriformis*.

It has previously been shown that 3 d P. aeruginosa biofilms coincubated with the surface feeding flagellate, *R. nasuta*, resulted in the death of the flagellate within 24 h (4). The grazing resistance of the P. aeruginosa WT biofilm was attributed to the formation of microcolonies and the *las* and *rhl* quorum sensing systems, which are known to regulate the expression of virulence factors (e.g., rhamnolipids and pyocyanins) (21). Results presented here demonstrate that single species biofilms of P. aeruginosa were also resistant to predation by *T. pyriformis*, as the biofilm biomass was unaffected by grazing (Fig. 1c, g, and k, and 2a). When P. aeruginosa was grown in combination with the grazing sensitive P. protegens and K. pneumoniae in a mixed species biofilm, all species in the biofilm were protected, indicating that P. aeruginosa provides associational resistance to other members of mixed species biofilms.

Previous studies have demonstrated that the presence of resistant species can confer cross-protection to sensitive species in the biofilm. For example, single species biofilms of P. aeruginosa and K. pneumoniae were sensitive to tobramycin (11). However, when grown with tobramycin resistant P. protegens, the mixed species biofilm was resistant against tobramycin, and there were no significant difference between the proportions of the two sensitive species in the control and treated biofilms (11). In contrast, a mixed species biofilm consisting of K. pneumoniae, P. protegens, and Staphylococcus epidermidis was sensitive to protozoan grazing by both A. castellanii and *Colpoda maupasi* (22). Here, it was demonstrated that P. aeruginosa may be a beneficial member in a mixed species biofilm under predation pressure, as *T. pyriformis* has been shown to be an effective predator of biofilms (23, 24) (Fig. 2a).

Cell-free supernatants obtained from P. aeruginosa biofilms were toxic to *T. pyriformis* (Fig. 2c), suggesting the presence of secreted antiprotozoal compounds. Rhamnolipids produced by P. aeruginosa have been shown to be necrotic factors responsible for lysing polymorphonuclear leukocytes (PMNs) (25, 26) and monocyte macrophages (27). Rhamnolipids have also been suggested to function as a “biofilm shield,” which significantly reduces the ability of PMNs to attack biofilm cells (26). It was reported that 0.01 g L^−1^ of purified rhamnolipids from P. aeruginosa resulted in the lysis of the amoeba, Dictyostelium discoideum, while supernatant obtained from a *rhlA* mutant strain did not cause lysis (28). Thus, we investigated the role of rhamnolipids produced by P. aeruginosa in providing overall biofilm grazing resistance.

Results here confirm that the production of rhamnolipids by P. aeruginosa biofilms is a defense mechanism against *T. pyriformis* grazing where 0.03 g L^−1^ of purified rhamnolipids (di-rhamnolipid dominant) was sufficient to kill *T. pyriformis*. Exposure of *T. pyriformis* to rhamnolipids resulted in membrane rupture of the protozoa, consistent with the proposed mechanism of action, membrane solubilization (Supplemental Video 1). Quantification of rhamnolipids from the supernatants of single and mixed species biofilms greatly exceeded the amount of rhamnolipids required to kill *T. pyriformis* (Fig. 5). P. protegens has not been shown to produce rhamnolipids; however, the orcinol extraction of the biofilm supernatant indicated that rhamnolipids or a similar type of compound were present. P. protegens produces a class of amphiphilic molecules known as cyclic lipopeptides (CLPs), which are similar to rhamnolipids. Hence, these CLPs may have been detected in the orcinol assay.

The supernatant from the mixed species biofilm was not as toxic as the supernatant from the P. aeruginosa biofilms, as there is 10-fold less rhamnolipid in the mixed biofilm (Fig. 2c and 5). Part of the reduction in rhamnolipids could be accounted for by the observation that P. aeruginosa represents only 40% of the community biomass. It is also possible that *rhlA* expression is repressed in the mixed species biofilm or the rhamnolipids are bound in the biofilm matrix. P. aeruginosa has been demonstrated to respond to the presence of PMNs by the upregulation of rhamnolipid synthesis (29). In contrast, there was no significant difference in the concentration of rhamnolipids produced by ungrazed and grazed biofilms, indicating that rhamnolipid production was not induced by the presence of *T. pyriformis*. Regardless, it is clear that production of rhamnolipid by P. aeruginosa plays a partial role in protecting the biofilm against *T. pyriformis* grazing.

Despite the lack of rhamnolipid production, the single species biofilms of P. aeruginosa *ΔrhlA* was resistant to *T. pyriformis* grazing, while the cell-free supernatant from the *ΔrhlA* mutant strain was nontoxic (Fig. 3c and 5). Protozoa were also observed to lyse within 5 min when added to a P. aeruginosa PAO1Δ*rhlA* biofilm (data not shown). Thus, we investigated the role of the type III secretion system (T3SS) in predation resistance. The T3SS is a contact-based defense system that has been shown to be involved in killing the amoeba A. castellanii and D. discoideum (14, 30). The T3SS forms complex needlelike structures on the bacterial cell surface that injects effector proteins into eukaryotic cells that come in contact with the bacterial cells (31). These effector proteins, ExoT and ExoU, are associated with rapid cell death of eukaryotic cells (31). Here, both mixed and single species biofilms containing a P. aeruginosa strain deficient in both T3SS and rhamnolipid production remained grazing resistant.

Although the total biovolume for all combinations of the mixed species consortia remained the same, the defect in *rhlA* resulted in a reduction in the ratio of P. aeruginosa from 39% in the mixed species WT to 22% and 13% for *ΔrhlA* and *ΔrhlA ΔpscJ* biofilms, respectively (Fig. 3a and 4a). Rhamnolipids are known to affect microbial adhesion and biofilm development (32). Inactivation of the *rhlA* gene would result in inhibition of swarming motility and a reduction in twitching motility (33), both forms of which are essential for the initial development of P. aeruginosa microcolonies (32). Furthermore, rhamnolipids also exhibit antibacterial properties (34). Thus, the lack of rhamnolipids may have affected the ability of P. aeruginosa to prevail in a mixed species biofilm. Furthermore, the decline in P. aeruginosa biomass resulted in an increase in K. pneumoniae biomass on the surface of the biofilm (Fig. S3). Therefore, it is possible that the localization of K. pneumoniae reduced the contact between P. aeruginosa and *T. pyriformis*, which resulted in lowered mortality of the predators.

An untargeted gene deletion through transposon insertion was also used to screen for additional mechanisms contributing to protozoa toxicity, and results revealed that the PQS pathway seemed to be involved in regulating toxin production, including PqsA, PqsB, PqsC, PqsD, and PqsR. The concentration of extracellular PQS in bacterial cultures has been reported to range from 6 μM to 30 μM with greater concentrations present in biofilms (35–37). PQS concentrations of up 2 μmol L^−1^ have been reported in the sputum samples of CF patients (38). At 2.5 to 5 μM PQS concentrations, there was no significant reduction in protozoa. The number of protozoa at low concentrations of PQS was reduced by 52% compared to the control in the absence of PQS with 1920 protozoa present. This could suggest that PQS might be inhibiting *T. pyriformis* proliferation as the protozoa numbers did not increase with 2.5 μM PQS. Studies have shown that PQS is also toxic to other bacteria and eukaryotes. Concentrations of PQS up to 50 μM have been reported to inhibit proliferation of blood mononuclear cells and T-cells (39, 40) and is also cytotoxic to airway epithelial cells and macrophages by inducing oxidative stress and altering the cell integrity and gap channel conductivity (41, 42). In addition, the growth of D. discoideum, a soil-dwelling amoeba, was inhibited at 1 μM PQS for 18 h (42). Thus, it seems that the PQS can inhibit protozoa growth and poses toxicity to the protozoa as the toxicity was concentration dependent, with 50 μM PQS being able to significantly reduced protozoa numbers. PQS also has antimicrobial activity and can suppress the growth of bacteria species, including S. aureus and *Vibrio* sp (43, 44). Moreover, it has been shown that the production of PQS by P. aeruginosa was lethal to Caenorhabditis elegans infection model at 40 μM (45). Vrla et al. (42) observed that PQS displayed concentration-dependent killing of monocytes from 0 to 50 μM after 18 h, similar to the toxicity of PQS toward *T. pyriformis* in this study.

Due to its hydrophobic nature, PQS interacts with the outer membrane lipopolysaccharidesand inserts into the cellular membrane, thus inducing membrane curvature to form outer membrane vesicles (OMVs) for trafficking of virulence factors and cell communication (46). It has been shown that within the biofilm the EPS was found to retain PQS (47, 48). OMVs are ubiquitous in biofilms and OMVs packed with PQS have been known to contribute to biofilm formation (49, 50). The OMVs were shown to promote PQS inhibition of S. aureus growth in a biofilm coculture formed by P. aeruginosa and S. aureus. To quantify quorum sensing signals, Charlton et al. (2000) compared different quorum sensing molecules in P. aeruginosa biofilm and noted that the relatively hydrophobic N-3-oxo-dodecanoyl-l-homoserine lactone accumulates to a higher concentration in the biofilm than the cellular fraction. Hence, the hydrophobicity and the packaging of PQS into OMVs. might have allowed PQS to accumulate in the biofilm to sufficient concentrations to be toxic. Similarly, in this study, the hydrophobic nature of might enable PQS to accumulate to concentrations sufficient to be toxic to the protozoa in the biofilm, while PQS concentrations in the aqueous phase were too low to kill the protozoa.

Upon PQS addition, toxicity was restored in P. aeruginosa Δ*rhlA*Δ*pqsA* transposon biofilm and partially in other P. aeruginosa Δ*rhlA* transposon biofilms, namely, *pqsB*, *pqsC*, *pqsD,* and *pqsR*. It is interesting to note that the *pqsA* mutant, which is the first gene in the operon, is more toxic to *T. pyriformis* than the tranpsoson mutants of the downstream PQS genes. LC-MS analysis confirmed that the *pqsA* mutant did not produce any detectable PQS (data not shown). Thus, while it is clear that PQS is one of the killing factors, further work is needed to better correlate the killing of *T. pyriformis* with each of the genes in the operon and the specific Tn insertions.

In conclusion, our results demonstrate that the presence of P. aeruginosa within the mixed species biofilm provides cross species protection for other members in the microbial community against protozoan grazing. Additionally, two toxicity mechanisms in P. aeruginosa were identified that affected *T. pyriformis*. The secretion of rhamnolipids, an amphiphilic glycolipid that can insert into cellular membrane to damage membrane integrity and lyse protozoa, acts as the primary protection mechanism against the protozoa. PQS was shown to act as the secondary toxicity factor. Together, these mechanisms allow the mixed species biofilm to survive protozoa predation, thus playing an important role in regulating interspecies interactions between community members and the internal balance between the species, highlighting the fact that diverse factors influence mixed microbial communities, and such defensive strategies can be shared among community members.

## MATERIALS AND METHODS

### Bacterial and protozoal strains.

Bacterial strains (Table 2) were cultured on Luria-Bertani (LB) agar with either 100 μg mL^−1^ gentamicin or 30 μg mL^−1^ tetracycline. Prior to inoculation for biofilm grazing assays, single colonies were inoculated into M9 minimal medium (48 mM Na_2_HPO_4_; 22 mM KH_2_PO_4_; 9 mM NaCl; 19 mM NH_4_Cl; 2 mM MgSO_4_; 0.1 mM CaCl_2_; and 0.04% wt/vol glucose) supplemented with 0.2% Casamino Acids and incubated for 24 h at 24°C. *T. pyriformis* was maintained axenically in PYG medium (with a per L composition of 20 g of peptone, 1 g of yeast extract and 50 mL of 2 M glucose) at 24°C.

**TABLE 2:** Strains and plasmids used in this study

Strains and plasmids	Relevant characteristic^*a*^	Source or reference
Bacteria		
P. aeruginosa PAO1	eYFP, Gm^R^	11
P. aeruginosa Δ*rhlA_gltA*-A2	Mariner C9 transposon, Gm^R^	This study
P. aeruginosa Δ*rhlA_gltA*-B2	Mariner C9 transposon, Gm^R^	This study
P. aeruginosa Δ*rhlA*_*gltA*-B10	Mariner C9 transposon, Gm^R^	This study
P. aeruginosa Δ*rhlA_gltA*-E9	Mariner C9 transposon, Gm^R^	This study
P. aeruginosa Δ*rhlA_gltA*-G12	Mariner C9 transposon, Gm^R^	This study
P. aeruginosa Δ*rhlA_*PA1417	Mariner C9 transposon, Gm^R^	This study
P. aeruginosa Δ*rhlA_*PA2228	Mariner C9 transposon, Gm^R^	This study
P. aeruginosa Δ*rhlA_pqsA*-G3	Mariner C9 transposon, Gm^R^	This study
P. aeruginosa Δ*rhlA_pqsB*-C2	Mariner C9 transposon, Gm^R^	This study
P. aeruginosa Δ*rhlA_pqsC*-A8	Mariner C9 transposon, Gm^R^	This study
P. aeruginosa Δ*rhlA_pqsC*-H8	Mariner C9 transposon, Gm^R^	This study
P. aeruginosa Δ*rhlA_pqsD*-E2	Mariner C9 transposon, Gm^R^	This study
P. aeruginosa Δ*rhlA_pqsR*-E9	Mariner C9 transposon, Gm^R^	This study
P. aeruginosa Δ*rhlA_pqsR*-E10	Mariner C9 transposon, Gm^R^	This study
P. aeruginosa Δ*rhlA_pqsR*-H1	Mariner C9 transposon, Gm^R^	This study
P. aeruginosa Δ*rhlA_pqsR*-H6	Mariner C9 transposon, Gm^R^	This study
P. protegens Pf-5	eCFP, Gm^R^	11
K. pneumonia KP-1	DsRedExpress, Gm^R^	11
P. aeruginosa Δ*rhlA*	eYFP, Gm^R^, Δ*rhlA*	59, 32
*P aeruginosa* Δ*rhlA*, *pscJ*	eYFP, Gm^R^, Tc^R^, Δ*rhlA*, Δ*pscJ*	This study
P. aeruginosa Δ*pscJ*	eYFP, Gm^R^, Tc^R^, Δ*pscJ*	This study
E. coli S17-1 λpir	*rec A thi pro hsdR* [RP4::2-Tc::Mu-Km::Tn7] λ pir Sm^R^ Tp^R^	V. L. Miller et al. 60
E. coli SM10 λpir	*rec A thi pro hsdR* [RP4::2-Tc::Mu-Km::Tn7] λ pir Km^R^	
DH5α	F-φ80*lac*ZΔM15 Δ(*lac*ZYA*arg*F)U169 *rec*A1 *end*A1 *hsd*R17(r_k_^−^, m_k_^+^) *pho*A *sup*E44 *thi*-1 *gyr*A96 *rel*A1 *ton*A	R. G. Taylor et al. 61
Protozoa		
*T. pyriformis*	Wild type, planktonic, and biofilm lifestyle	CCAP/1630/1F; Culture Collection of Algae and Protozoa, UK
Plasmids		
pEX18ApGW	Ap^R^, Gm^R^	15
pK18mobsacB	*sacB*, Gm^R^	62
pBT20	Ap^R^, Gm^R^	63

a Ap^R^, ampicillin; Gm^R^, gentamicin; Km^R^, kanamycin; Sm^R^, streptomycin; Tc^R^, tetracycline; Tp^R^, trimethoprim.

### Construction of P. aeruginosa type III secretion mutants.

To generate a mutant lacking the type III secretion system, *pscJ* was deleted using allelic exchange (51). Flanking regions of the *pscJ* gene of P. aeruginosa were amplified by PCR, and *tetA* was amplified using pBR322 as the template. The three PCR fragments were fused by second-round SOE PCR using primers with overhangs, introducing SacI and *Xbal* restriction sites. The SOE PCR product was introduced into pEX18ApGw digested with SacI and X*bal* (15). The pEX18Ap-Δ*pscJ*::*tet* plasmid was then transformed into E. coli S17-1 λpir and subsequently introduced into P. aeruginosa PAO1_YFP_ and PAO1_YFP_ Δ*rhl* (52). The resulting *pscJ* deletion mutants were selected on M9 medium supplemented with 0.2% glucose and 100 μg mL^−1^ tetracycline and LB medium supplemented with 100 μg mL^−1^ tetracycline. The *pscJ* deletion mutants were verified by Sanger Sequencing.

### Construction of P. aeruginosa quorum sensing and secretion systems mutants.

Deletion constructs were assembled with NEBuilder HiFi DNA Assembly (New England Biolabs, USA) with deletion primers (Table 3). All amplifications were carried out using Q5 polymerase (New England Biolabs, USA), with PCR products analyzed by DNA agarose electrophoresis (1% wt/vol) and purified by Wizard SV gel and PCR Clean-Up System (Promega, USA). The pK18mobsacB-Gm plasmid was used for markerless gene deletion and amplified with primers flanking HindIII and PstI restriction sites: forward, 5′ aagcttggcactggccgt 3′ and reverse, 5′ cctgcaggtcgactctagag 3′. DNA sequences flanking the target gene were inserted into the plasmid and amplified in E. coli DH5α cells via heat shock before being extracted using FavorPrep plasmid extraction kit (Favorgen Biotech, Taiwan). Inserted sequences were verified by Sanger sequencing (Biobasic Asia Pacific, USA). Mutants were generated in the P. aeruginosa Δ*rhlAB* background by electroporating deletion plasmids into the bacteria and spread on LB agar containing gentamicin to screen for single crossover clones. Mutants were then grown at 37°C with shaking (200 rpm) in LB medium without any antibiotics for 6 h. To select mutants in which the second recombination had occurred, the cultures were diluted 10× and spread on LB agar containing 12% sucrose (wt/vol) and grown overnight at 37°C. Single colonies were patched onto LB agar and LB agar containing gentamicin simultaneously. Colonies sensitive to gentamicin were verified by colony PCR and sanger sequencing.

**TABLE 3:** Primers

Amplicon	Primer	Sequences 5′–3′
5′, upstream region of *pscJ*	pscJ::tetRA1	GCAGCAAACCTTTCTCCTCC
	pscJ::tetRA2	GGAGAACTGTGAATGCGCATGGGTCTTCATCAGGGTTTC
3′, downstream region of *pscJ*	pscJ::tetRB1	TCTTGAAGACGAAAGGGCCTATGCCATTGACGGCCTAC
	pscJ::tetRB2	CGCCGGAAATAGCCTTCTT
*tet* from pBR322	pscJ::tetR_tet1	GAAACCCTGATGAAGACCCATGCGCATTCACAGTTCTCC
	pscJ::tetR_tet2	GTAGGCCGTCAATGGCATAGGCCCTTTCGTCTTCAAGA
Adds overhangs to the *tet* amplicon	pscJ::tetR_SOE_XbaI	CTACTATCTAGAACCTTTCTCCTCCAGTTGCT
	pscJ::tetR_SOE_SacI	CTATCTGAGCTCGGAAATAGCCTTCTTCCGG
Test for insertion into the genome	pscJ::tetR_checkA	AACGGGTGCGCATAGAAAT

### Biofilm grazing assays.

Grazing assays were established in 24-well microtiter plates as described in Matz et al. (2004) with modifications. Bacterial strains grown for 24 h at 24°C were adjusted to approximately 1 × 10^8^ CFU mL^−1^. Single species biofilms were established by inoculating microtiter plates wells with a 1:10 dilution of the bacterial strain in 1 mL of M9. Mixed species biofilms of P. aeruginosa wild type or mutant strains, P. protegens and K. pneumoniae were established by mixing the cultures at a ratio of 5:5:1 to account for the higher growth rate of K. pneumoniae (11). Biofilms were grown at 24°C for 48 h with gentle shaking (50 rpm). Spent medium was replaced with fresh M9 medium after 24 h in both single and mixed species biofilms, and the biofilms were incubated for another 24 h before *T. pyriformis* was added at a final concentration of 10^3^ cells mL^−1^. Controls for the biofilm assays contained M9 medium with heat killed bacteria (HKB) added at a cell density of 10^8^ cells mL^−1^ and *T. pyriformis* at a final cell density of 10^3^ cells mL^−1^. Ungrazed and grazed biofilms were prepared in duplicate. Biofilms were examined with confocal laser scanning microscopy (CLSM) (LSM 780, Carl Zeiss, Germany) to observe the effects of grazing after 48 h. For quantification of *T. pyriformis*, triplicates of 10 μL were removed from each well and the numbers of *T. pyriformis* determined using light microscopy (Primo Star, Carl Zeiss, Germany).

### Supernatant toxicity assay.

Cell-free supernatants were obtained from ungrazed and grazed biofilms by filtration (0.22 μm) and 700 μL added to 24-well microtiter plates containing *T. pyriformis* at 10^3^ cells mL^−1^ in 1 mL of M9. Controls for the supernatant assays were *T. pyriformis* at a final cell concentration of 10^3^ cells mL^−1^ in M9 medium. *T. pyriformis* was enumerated by light microscopy after 24 h.

### Rhamnolipid toxicity assay.

The toxicity of purified rhamnolipids (95% di-rhamnolipid; Technologies, USA) (0.01 to 0.5 g L^−1)^ to *T. pyriformis* (10^3^ cells mL^−1^) was tested in 96-well microtiter plates. Controls consisted of 10^3^ cells mL^−1^ of *T. pyriformis* in M9 media. Both controls and treatments were prepared in triplicate with a working volume of 100 μL each. Ciliates were enumerated after 24 h using light microscopy. Inactive *T. pyriformis* were considered to be dead.

### Rhamnolipid quantification.

The concentration of rhamnolipids from the single and mixed species biofilms were determined by the orcinol assay as previously described (53). Supernatants from 48 h grazed and ungrazed biofilms were filtered (0.22 μm) and 0.5 mL was extracted twice with 2 volumes of diethyl ether. The ether fractions were evaporated until dry and reconstituted in 100 μL dH_2_O. For each 100 μL sample, 100 μL of 1.71% orcinol solution, and 800 μL of 60% (vol/vol) H_2_SO_4_ were added. The samples were heated at 80°C for 30 min, cooled for 15 min at 24°C, and the absorbance (A_421 nm_) was measured and compared to rhamnose standards (0 to 40 g L^−1^). Rhamnolipid concentrations were determined as 1.0 mg of rhamnose corresponds to 2.5 mg of rhamnolipid (21, 54).

### Generation of a P. aeruginosa ΔrhlA transposon library.

The mariner transposase vector, pBT20, was used in E. coli SM10-*λpir* for biparental conjugation with the P. aeruginosa Δ*rhlA* strain. E. coli and P. aeruginosa were grown in LB overnight with appropriate antibiotics (200 rpm at 37°C). One hundred microliters of P. aeruginosa and SM10-*λpir* were spread onto LB plates and LB plates with 30 μg mL^−1^ gentamicin, respectively, and incubated overnight at 37°C. Bacterial lawns from both the E. coli donor and the P. aeruginosa Δ*rhlA* recipient were then scraped off the plates and mixed thoroughly to form a conjugation mix. The process was repeated to obtain 3 conjugation mixes. Each conjugation mix was incubated on the center of an LB agar plate for 2 h. After incubation, all conjugation mixes were resuspended together in LB to a final volume of 15 mL and aliquoted into cryotubes of 1 mL for frozen stock. Fifty microliters of the resuspension was plated on Vogel-Bonner Medium (VBM) (0.8 mM MgSO_4_, 0.96 mM citric acid, 0.17 mM K_2_HPO_4_, 2.27 mM NaNH_5_PO_4_, pH 7) with 1.5 % agar (wt/vol) containing 80 μg mL^−1^ gentamicin to select for P. aeruginosa Δ*rhlA* transposon mutants. Colonies of putative mutants were individually inoculated in separate wells containing 100 μL of LB in 96-well microtiter plates and grown for 6 h (200 rpm at 37°C). After 6 h, wells with growth were transferred into a new 96-well microtiter plate and mixed with an equal volume of LB containing 20% (vol/vol) glycerol to be kept as frozen stock, producing a library of approximately 4,000 mutants.

Subsequently, each mutant in the P. aeruginosa Δ*rhlA* transposon library was screened for the loss of protozoa toxicity using the grazing assays described above. Genomic DNA was extracted from each mutant using QIAamp DNA minikit (Qiagen, Netherlands) according to manufacturer protocols. Mutants that allowed for protozoan growth (nontoxic) were sent for genome sequencing by Miseq 300 bp paired end sequencing (Illumina, USA). The sequencing data were quality checked using FastQC (55), trimmed to remove adaptors with Fastp (56), and analyzed by Breseq (57) for the genome location of the transposon insertion.

### PQS quantification by LC-MS.

PQS was extracted from P. aeruginosa Δ*rhlA* biofilm following the protocol established by G. C. Palmer et al. (58). P. aeruginosa Δ*rhlA* biofilms, as grown in grazing assays, were pooled from 24 wells after 2 d and mixed with an equal volume of acidified ethyl acetate (0.15 mL L^−1^ glacial acetic acid) to collect the organic phase. The process was repeated twice to fully extract PQS. Subsequently, the ethyl acetate extract was dried in 50 mL falcon tubes in a CentriVap benchtop vacuum concentrator (Labconco, USA) at 26°C for 6 h. Dried extracts were resuspended in 50 μL of methanol (HPLC grade) and transferred to 1 mL Eppendorf tubes for storage in dark at –20°C. PQS standards were prepared by dissolving PQS (Sigma-Aldrich, USA) in methanol (HPLC grade).

Resuspended extracts were characterized by targeted liquid chromatography-mass spectrometry (LCMS) analysis to quantify the amount of PQS (Singapore Phenome Centre, Lee Kong Chian School of Medicine, NTU). A total of 40 μL of LCMS grade methanol was added to 10 μL of the resuspended extracts and mixed using vortex. The samples were then incubated at 4°C for 15 min, followed by centrifuging at 14,000 g at 4°C for 10 min. The supernatant was collected and injected into a waters BEH C18 (2.1 mm × 100 mm, 1.7 μm) column with the mobile phase A of 0.1% (vol/vol) formic acid in water and the mobile phase B of 0.1% (vol/vol) formic acid in acetonitrile. The samples were separated using a 50% phase A and B for 30 s, a linear 50% phase A to 100% phase B for 90 s, kept at 100% phase B for 2 min, returned to 50% phase A in 6 s, and stabilized for another 3 min. The HPLC flow rate was 0.5 mL min-1, and the column temperature was kept at 40°C. The samples were introduced into the mass spectrometer (Waters Xevo TQ-S) for positive electrospray ionization analysis, using a capillary voltage of 3 kV and a source temperature of 150°C. PQS was quantified in a multiple reaction mode. A quality control sample was prepared by pooling the extracted samples into one mixture and analyzed along with the samples to ensure method reliability and instrument stability.

### Biofilm formation assays of P. aeruginosa ΔrhlA transposon mutants.

To measure biofilm formation, overnight bacterial cultures were normalized to OD600 1.0 before being further diluted 1:50 in fresh medium. One hundred microliters of the diluted bacterial cultures were added to 96-well microtiter plates (Nunclon Delta-treated polystyrene, Thermo Scientific, USA). Plates were incubated at 37°C without agitation and biofilm formation was quantified by crystal violet staining after 6 h. Supernatant was removed from each well and washed with 150 μL of phosphate-buffered saline (PBS). The plate was stained with 125 μL of 0.1% (vol/vol) crystal violet for 15 min. The crystal violet was then removed, and each well was washed twice with 150 μL of PBS before adding 200 μL of absolute ethanol. One hundred microliters of the absolute ethanol was transferred into a clean plate for quantification at OD570.

### Microscopy, image, and statistical analyses.

Image acquisition was performed on a CLSM (LSM 780, Carl Zeiss) using the multitrack mode. Excitation wavelengths for eCFP, eYFP, and DsRedExpress were 458, 514, and 561 nm, respectively, while the emission wavelengths were 476, 527 and 584 nm, respectively. Image stacks were obtained from each well, starting from the center and 2.0 mm toward the top and bottom. Quantification of confocal micrographs was performed using Imaris (Bitplane AG, Belfast, UK) to obtain values for the volume and area of the biofilms. One-way ANOVA was performed using Prism (GraphPad 6.0) to determine the effectiveness of protozoan grazing on biomass reduction in biofilms.
