# Supplementary material for: Associational Resistance to Predation by Protists in a Mixed Species Biofilm
Source: Appl Environ Microbiol. 2023 Jan 19;89(2):e01741-22. doi: 10.1128/aem.01741-22 (PMC9972941; doi:10.1128/aem.01741-22)
Supplement: Supplemental file 1 — Fig. S1 to S4. Download aem.01741-22-s0002.pdf, PDF file, 0.3 MB [file aem.01741-22-s0002.pdf]

Supplementary material

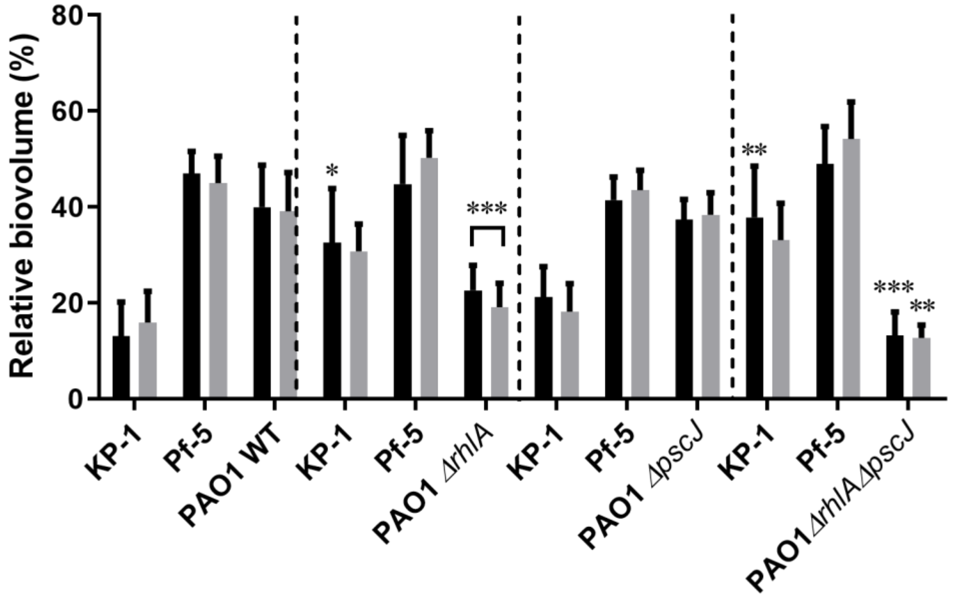

FIG S1. Relative percent biovolume of *P. aeruginosa* PAO1, *P. protogens* Pf-5 and *K. pneumoniae* KP-1 in the mixed species ungrazed (black bars) and grazed (grey bars) biofilms after 48h of grazing by *T. pyriformis* (n=3). Biovolumes were determined by image analysis of images collected by confocal microscopy. Biovolume was tested for each bacterial species against the wild type for grazed and ungrazed biofilms. * <0.05, ** P<0.01, *** P <0.001.

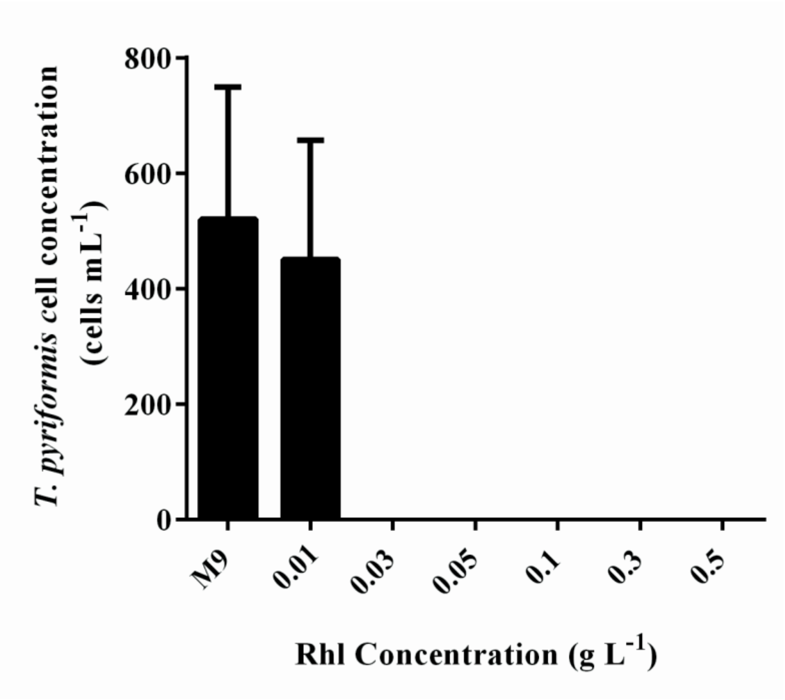

FIG S2. Rhamnolipid mediated killing of *T. pyriformis.* A minimum concentration of 0.03 g L^{-1} was found to be effective in lysing the ciliate (n=3).

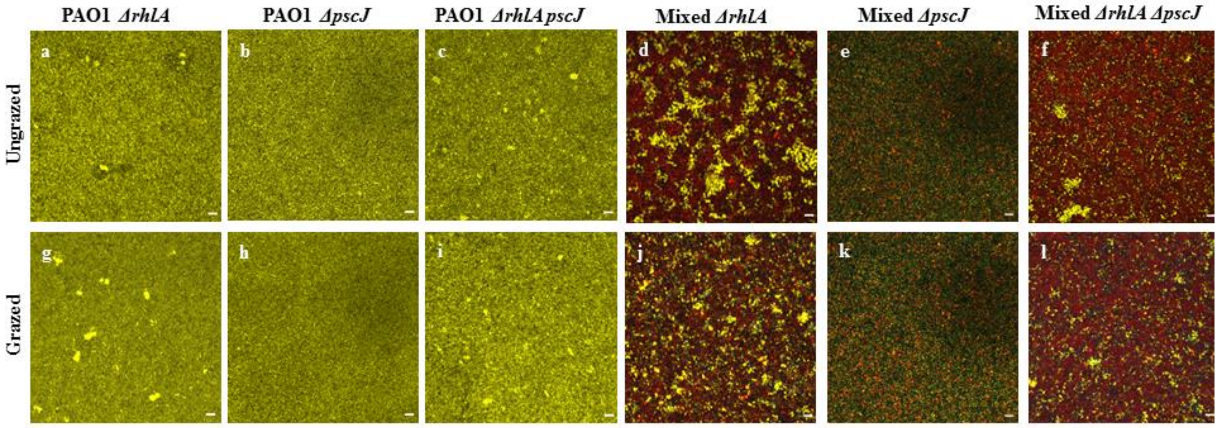

FIG S3. Confocal micrographs of ungrazed and grazed single and mixed species biofilms containing the wild type *P. aeruginosa* or *P. aeruginosa ΔpscJ*, *ΔrhlA* or *ΔrhlA ΔpscJ* in the presence and absence of *T. pyriformis*. Biofilms were pre-grown for 48 h, at which time, either nothing was added (non-grazed controls, gray bars) or *T. pyriformis* was added and the biofilms were incubated for a further 48 h (96 h total). (a to l). These panels compare the difference in biomass between the ungrazed and grazed single and mixed species biofilm. Magnification: scale bars: 20 µm. *P. aeruginosa* (yellow), *P. protegens* (blue) and *K. pneumoniae* (red).

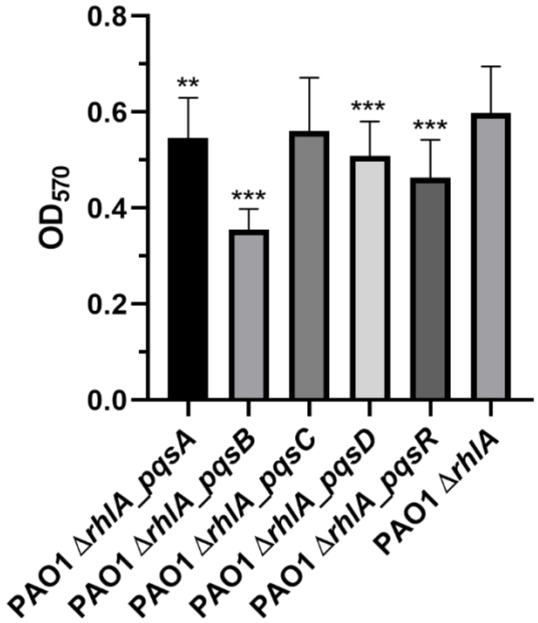

FIG S4. Biofilm quantification of *P. aeruginosa* Δ*rhlA* and transposon mutants after 6 h of growth measured by crystal violet staining. Data points represent the mean ± standard deviation of the mean (SD) (n=3). ** $P<0.01$, *** $P<0.001$.
